# Repurposed 3D Printer Allows Economical and Programmable Fraction Collection for Proteomics of Nanogram Scale Samples

**DOI:** 10.1021/acs.analchem.4c01731

**Published:** 2024-07-05

**Authors:** Eduardo S. Kitano, Gareth Nisbet, Yana Demyanenko, Katarzyna M Kowalczyk, Louisa Iselin, Stephen Cross, Alfredo Castello, Shabaz Mohammed

**Affiliations:** † Rosalind Franklin Institute, Harwell Campus, Didcot OX11 0QX, United Kingdom; ‡ Department of Pharmacology, University of Oxford, Oxford OX1 3QT, United Kingdom; § Diamond Light Source, Harwell Science and Innovation Campus, Didcot OX11 0DE, United Kingdom; ∥ MRC-University of Glasgow Centre for Virus Research, Glasgow G61 1QH, United Kingdom; ⊥ Nuffield Department of Medicine, Peter Medawar Building for Pathogen Research, University of Oxford, Oxford OX1 3SY, United Kingdom; # Department of Biochemistry, University of Oxford, Oxford OX1 3QU, United Kingdom; ∇ Department of Chemistry, University of Oxford, Oxford OX1 3TA, United Kingdom

## Abstract

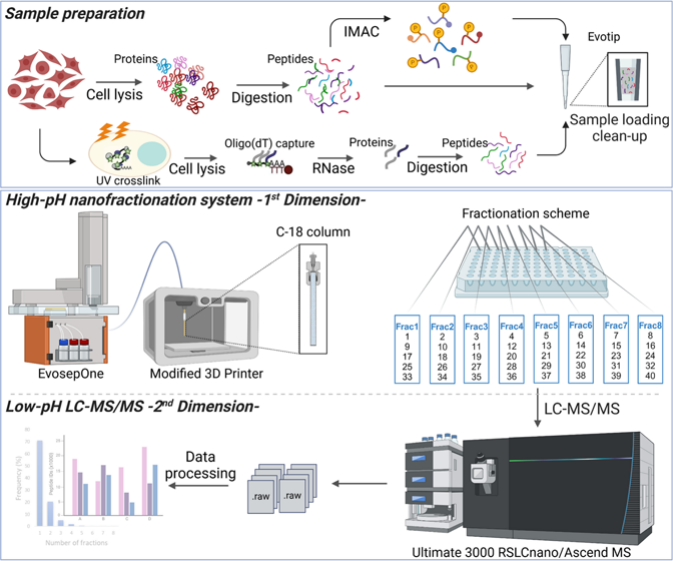

In this work, we describe the construction and application of a repurposed 3D-printer as a fraction collector. We utilize a nano-LC to ensure minimal volumes and surfaces although any LC can be coupled. The setup operates as a high-pH fractionation system capable of effectively working with nanogram scales of lysate digests. The 2D RP–RP system demonstrated superior proteome coverage over single-shot data-dependent acquisition (DDA) analysis using only 5 ng of human cell lysate digest with performance increasing with increasing amounts of material. We found that the fractionation system allowed over 60% signal recovery at the peptide level and, more importantly, we observed improved protein level intensity coverage, which indicates the complexity reduction afforded by the system outweighs the sample losses endured. The application of data-independent acquisition (DIA) and wide window acquisition (WWA) to fractionated samples allowed nearly 8000 proteins to be identified from 50 ng of the material. The utility of the 2D system was further investigated for phosphoproteomics (>21 000 phosphosites from 50 μg starting material) and pull-down type experiments and showed substantial improvements over single-shot experiments. We show that the 2D RP–RP system is a highly versatile and powerful tool for many proteomics workflows.

## Introduction

Technological advancements in liquid chromatography (LC)^1−4^ and mass spectrometry (MS)^5−7^ have significantly improved the depth of proteome coverage in bottom-up proteomics. The combination of these two analytical tools on the analysis of peptides from cells or tissues has become the state-of-the-art workflow in proteomics. New mass spectrometry configurations^8,9^ combined with robust nano-LC^10,11^ have led to a considerable increase in proteome coverage and throughput. Nevertheless, deep and comprehensive proteome characterization by single-shot LC-MS still poses a challenge. Two-dimensional (2D) separation strategies play a pivotal role in obtaining a more complete characterization of complex biological samples by reducing the sample complexity.^12^

Several fractionation strategies have been used in combination with LC-MS/MS. Cation exchange^13−15^ and reversed-phase (RP)^16−19^ have been the most commonly used multidimensional separation modes, as reviewed by Yuan et al.^12^ High pH has proven popular due to the ease of setup and the use of concatenation to improve orthogonality with the second dimension low-pH RP nano-LC separation.^17^ Moreover, high- and low-pH 2D separation (2D RP–RP) benefits from the higher resolving power, compatible solvents between the first and second dimensions, and no need of desalting steps prior to the second dimension.

Despite the ability to unravel complex biological mixtures, a key limitation of 2DLC methodologies lies in the sample losses accrued when transferring between dimensions. Nonspecific binding of peptides to silica, metal, and plastic surfaces significantly contributes to peptide loss, compromising sensitivity and quantitative accuracy in LC-MS analysis. These sample losses restrict the use of 2D separations in studies where abundant material is readily available. For studies involving samples with limited availability, such as clinical specimens, rare cell populations, or those enriched for specific proteins and/or peptides, achieving adequate analytical depth while minimizing sample losses presents a significant challenge. Sample preparation approaches through the use of detergents have tried to minimize losses.^19,20^ Also, others have developed nano/microscale automated fractionator systems^18,21^ in an attempt to minimize losses during the transfer step of the fractions to second dimension separation, which can also include the use of low-binding containers, low surface area glass nanowell chips, and MS-compatible additives in downstream sample handling steps.^19,21,22^

A major challenge of a fractionation system is the need for bespoke or costly fractionation equipment. In this work, we built a fractionation system from an inexpensive 3D printer and a Raspberry Pi that is highly versatile and capable of fractionating small peptide amounts from cell lysates.

## Experimental Section

Detailed methods are provided in the Supporting Information.

### Sample Preparation and Protein Digestion

Expi293F cell (Gibco) lysate was submitted to in-solution LysC/trypsin digestion as previously described,^15^ while HeLa cell lysate was submitted to on-bead digestion using a solid-phase enhanced sample-preparation (SP3) method.^23^

### Phosphopeptide Enrichment

The desalted peptide sample from the digestion of HeLa cell lysate was submitted to the phosphopeptide Zr-IMAC enrichment protocol as previously described.^24^

### RNA Interactome Capture and Sample Preparation

RNA-binding proteins (RBPs) present in HEK293 cells were profiled via the RNA interactome capture (RIC) approach.^25,26^ After treating with benzonase, RIC samples were processed via the SP3 cleanup method followed by on-bead digestion as described before. For details, see the Supporting Information.

### Off-Line High-pH Reversed-Phase Nanofractionation System

Peptide samples were loaded on Evotip Pure and submitted to high-pH chromatography using an Evosep One system (Evosep Biosystems). Separation was performed in an in-house packed C-18 column and analyzed using the 30SPD method at 500 nL/min. Mobile phases A and B consisted of 10 mM triethylammonium bicarbonate (TEAB, pH8) and 100% ACN, respectively. Forty fractions were collected and concatenated into 8 main fractions in a 96-well plate at 1 min intervals using a 3D-printer-based fraction collector, fabricated from a Creality Ender 5 S1 printer, and powered by a Raspberry Pi computer (details in https://github.com/garethnisbet/Fraction-Collection-Unit and Supporting Information). For the peptide recovery experiment, fractions were manually combined into a single well to determine the sample loss.

### Nano-Liquid Chromatography, Mass Spectrometry, and Data Processing

Peptides were separated on an Ultimate 3000 RSLCnano system (Thermo Fisher Scientific) using an in-house packed 50 μm ID x 50 cm L Reprosil-Gold C-18 (Dr. Maisch) analytical column at 100 nL/min. Eluting peptides were electrosprayed into an Orbitrap Ascend Tribrid mass spectrometer (Thermo Fisher Scientific) using data dependent (DDA), data independent (DIA), or wide window acquisition (WWA) modes.

LC-MS/MS data were analyzed by four different platforms using the same human proteome database (Uniprot, proteome ID: UP000005640, downloaded in August 2022, 79759 sequences). DDA data sets were processed with the FragPipe computational platform with MSFragger.^27,28^ We opted to use MaxQuant^29^ for processing the DDA raw data from the phosphopeptide-enriched samples, the peptide recovery experiments, and also the chromatographic peak fwhm determination. DIA experiments were analyzed by DIA-NN software^30^ using library-free search, and WWA experiments were processed by INFERYS^31^ rescoring and CHIMERYS, implemented in Proteome Discoverer 3.0 software (Thermo Fisher Scientific). All experiments used standard parameters and were filtered to 1% FDR protein and peptide level. The LC and MS method details are described in Supporting Information. Raw files and results from this study have been deposited to the ProteomeXchange Consortium via the PRIDE^32^ partner repository with the data set identifier PXD051148. Subsequent analysis of data was performed in the Perseus environment^33^ and GraphPad Prism.

## Results and Discussion

### Designing a Robust High-pH Nano-LC System

**Figure 1:**
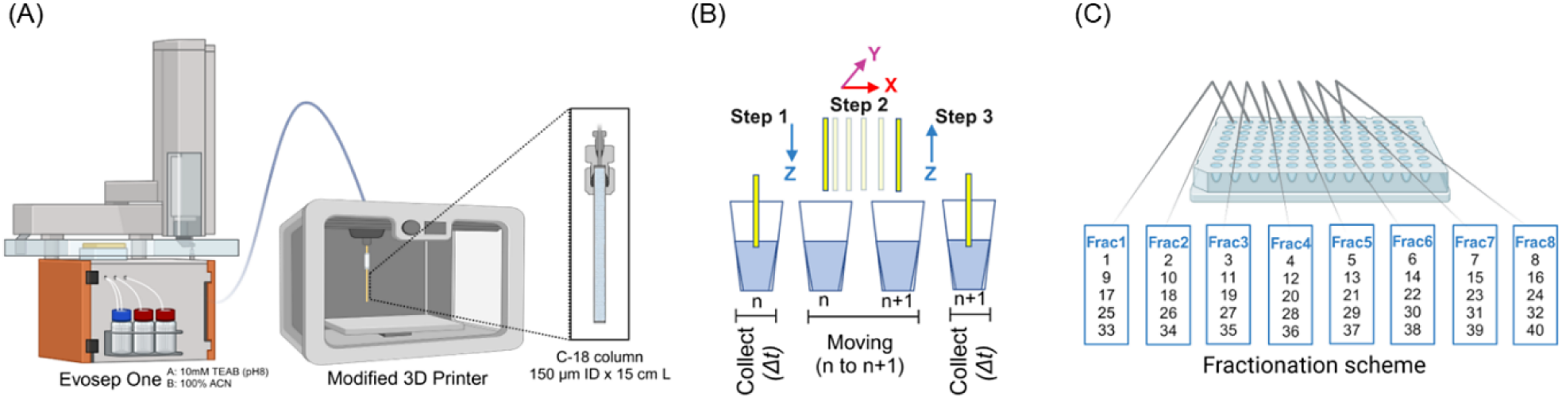
Off-line high-pH reversed-phase nanofractionation system overview. (A) Peptide samples were submitted to first dimension separation using an Evosep One HPLC system equipped with an in-house packed 15 cm L × 150 μm ID C-18 column coupled to a 3D-printer-based fraction collector. (B) XYZ moving mechanism of the fraction collector (position *n* to position *n* + 1), showing the desired column outlet position. (C) Fractionation scheme showing the combination of 40 fractions, at 1 min intervals, into eight concatenated main fractions (8 × 5 scheme). After first dimension separation, plates are inserted into the autosampler for the second dimension low-pH separation.

Nanolitre flow rate fraction collection systems require dedicated low sample loss instrumentation. We hypothesized that a cheap 3D printer that contains sufficient control in 3 dimensions is capable of being converted into a fraction collector (Figures 1A and S1A). The total cost of a 3D printer, including the controller and screen, was approximately 500 GBP. We dismantled the printer head in the 3D printer and developed a new controller that is powered by a Raspberry Pi computer. The software is written in Python and uses PyGame for the GUI, Numpy for mathematical operations, and PySerial for communication over USB. For the source code and detailed explanations, refer to the project’s GitHub repository: https://github.com/garethnisbet/Fraction-Collection-Unit. Custom 3D printed components were manufactured and adapted to the system for attaching the column and supporting the collection plate (Figure S1B,C). The system was designed to be flexible and able to automatically collect and concatenate multiple fractions into the 0.2 mL wells of a 96-well plate or 96-Evotip rack, depending on the LC system used for the second-dimension separation (Figure S1C). Due to the envisaged low volume expected from the LC, the effluent was collected by submerging the column outlet (∼1 mm) into approximately 50 μL of preloaded LC buffer (Figure 1B,C).

Although the fraction collector can be coupled to any LC, our use case was low sample amounts, and so a nano-LC would be the best choice due to its use of minimal surface areas and volumes. We hypothesized that the Evosep One could be a robust platform for nano-LC fractionation since the trap column is single use and elution is only up to 40% acetonitrile, eliminating the transfer of the more hydrophobic junk on to the analytical column. These two features substantially improve the robustness, has established Evosep One as a formidable choice for high throughput proteomics,^10,34^ and will allow low intervention fractionation. To convert this system for fractionation, we need to assess the system’s ability to operate at a basic pH as this is now considered optimal for the first dimension of a 2D RP–RP system. The separation system is centered on the use of a disposable trap column, where peptides are separated and stored in a loop by the gradients formed by four low-pressure pumps, and then this preformed gradient is pushed toward the analytical column for separation by a single high-pressure pump.^10^ The need for preforming gradients requires careful control of flow rates and pressures, and so the system only offers preset optimized methods. The addition of salt to the mobile phase can lead to salt precipitation in gradient separation as the acetonitrile fraction increases.^35^ First, we evaluated the performance of the LC using an in-house constructed 150 μm ID C-18 column operating at 500 nL/min (matching commercial option) at standard pH and pH 8. At pH 8, the backpressures generated by the system, irrespective of the solvent composition, were similar to standard conditions, indicating that the addition of TEAB to the mobile phase is not an issue (Figure S2A). Using the 30 SPD method resulted in a median peak width of 47 s at baseline, across a 44 min gradient (Figure S2B). Repeatability was similar to low pH, indicating that the analytical column is not affected by the mildly basic pH (Figure S3). When inspecting the retention time of peptides using a scatter plot from human cell lysate digestion that was separated using high and low pH, we observed a very loose correlation, the expected trend established by others (Figure S4).^16,36^ The peak width and gradient length will allow forty 1 min fractions and allow 5 concatenations and 8 highly discrete fractions, potentially allowing an order of magnitude increase in the peak capacity over a single dimension system. Similar 2D nano-LC RP–RP approaches using similar columns came to similar conclusions.^18,19^ The LC system easily transitions between regular nano-HPLC and fractionation modes, requiring approximately 1 h to perform a full cycle of solvent exchange, which confers the system flexibility and operational efficiency.

### Evaluating Sample Losses

To analyze potential sample losses associated with fractionation, 100 ng of peptides from the Expi293F digest resuspended in 5% FA were submitted to fractionation, and then the total eluted volumes were recombined. Half of the 100 ng fractionated/recombined sample was submitted to LC-MS/MS analysis, and the intensities of peptides were compared to the intensities of the same set of peptides quantified in the analysis of 50 ng of the nonfractionated sample. We observed that our 2D system was able to recover, on average, 74% (*N* = 3) of peptide in comparison to the unfractionated sample based on total intensity (Figure S5A). We also evaluated the effect of MS-compatible surfactants on the peptide recovery. It has been shown that the use of small concentrations of *n*-dodecyl-β-d-maltoside (DDM) significantly reduces peptide losses due to surface adsorption.^19^ We then repeated the experiment, resuspending the samples in 5% FA/0.015% DDM solution, followed by fractionation. We found that the use of DDM increased recovery to a level that suggested losses were negligible or within the quantitation error of the label free analysis (*N* = 3) for this highly controlled “high amounts” experiment (Figure S5B).

### Evaluating Fractionation Approach

**Figure 2:**
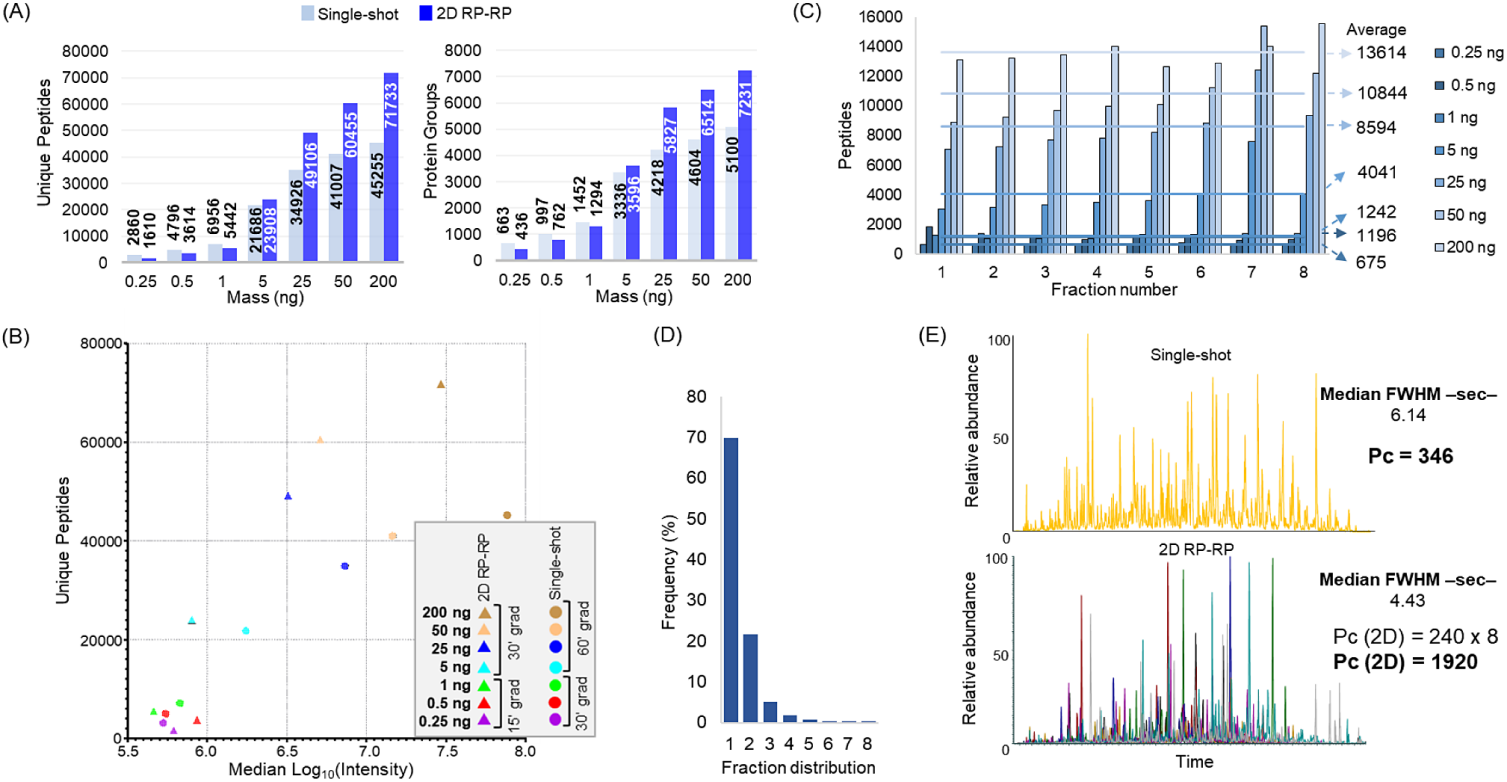
Performance of the 2D RP–RP at different human cell lysate digest sample amounts. (A) Number of unique peptides and protein groups identified in single-shot and 2D RP–RP applied to 0.25–200 ng sample amounts. (B) Number of unique peptides plotted as a function of median peptide intensities in single-shot and 2D RP–RP experiments according to the peptide sample amount. (C) Number of identified peptides across fractions. Numbers on the right indicate the average number of peptides identified per fraction according to the injected mass. (D) Histogram showing the frequencies of detected peptides in one or multiple fractions for 50 ng fractionation. (E) Base–peak chromatograms of the single-shot and 2D RP–RP showing the median values of fwhm and the theoretical peak capacity (Pc) values of single-shot and 2D RP–RP. Raw files were processed using Fragpipe or MaxQuant (for peak width calculation).

Having established a fractionation protocol, we then aimed to evaluate the performance and relative sensitivity of the 2D setup compared with a single-shot analysis. We opted to perform one “large scale” (400 ng) fractionation and then use it to test a range of sample amounts varying from 0.25 to 200 ng of Expi293F digest. Table S1 lists the identified peptides categorized by sample amount. To maximize the MS signal intensity and improve the detection and selection of peaks for fragmentation, we employed different gradient times. For single-shot, we employed gradients of 30 min for samples below 1 ng and for 60 min for the rest. For fractionated samples, we preferred 15 min for the analysis of peptide amounts below 1 ng (8 fractions, total 2 h gradient time) and 30 min for the rest (total 4 h). As expected, increasing sample amounts led to an increased number of unique peptides and protein groups in both single-shot and 2D RP–RP analyses. Below 1 ng of the sample, neither approach showed to be clearly superior although single-shot analyses potentially showed more favorable results (Figure 2A,B). In the lowest amount tested (0.25 ng), single-shot analysis resulted in 2860 unique peptides and 663 protein groups, corresponding to 78% and 52% more IDs than obtained by 2D. Above and including 5 ng, the fractionation system produced more identifications. Initially, the 5 ng fractionation resulted in the identification of 23 908 unique peptides and 3596 protein groups, representing increases of 10% and 8% compared to the single-shot analysis. Further increases in the sample amount resulted in a progressive relative gain (Figure 2A) in the identification of 2D over single-shot analysis. At 200 ng, 71 733 unique peptides and 7231 proteins were identified, reflecting substantial improvements of 58% and 42% in comparison to single-shot. Important to note, single-shot identifications exhibited a tendency to plateau once past 25 ng of the material, while in 2D, we did not observe such a leveling off for the amounts we applied, suggesting we will continue seeing improved results with higher amounts of material. The Evosep loading tips are capable of handling up to 1 μg. It is noteworthy that 2D experiments produced higher peptide identifications at a lower average peptide intensity demonstrating the value of complexity reduction (Figure 2B). The fractionation approach resulted in a similar number of identified peptides across the fractions (Figure 2C), with most of the peptides (70%) identified in one fraction (Figure 2D). Applying “match between runs” to the same data set reduced the number found in one fraction to 64% (Figure S6); a result similar to Kulak et al.^18^ even though column dimensions differ slightly (75 μm to our 150 μm, 300 nL/min to our 500 nL/min) and confirming our choice of concatenation parameters. Comparing a single-shot analysis chromatogram to a collection of chromatograms for the associated fractionation sample shows a similar overall profile with the 2D experiment showing more peaks that are sharper indicating the superior separation power. The 2D experiment resulted in an upper theoretical peak capacity value of 1920, representing approximately 6 times improvement in comparison to our single-shot experiment and exceeding any single-shot analysis performed (Figures 2E and S7).^37,38^ Although we opted for a concatenation approach, the advent of ultrarapid mass analyzers allows very short gradients (<5 min) and Guzman et al.^39^ have shown that nonconcatenated fractionation can be orthogonal, powerful, and simpler. Our fractionation system is compatible with this 2D setup, as we can change the fractionation collection pattern.

### Performance at Low Sample Amounts

**Figure 3:**
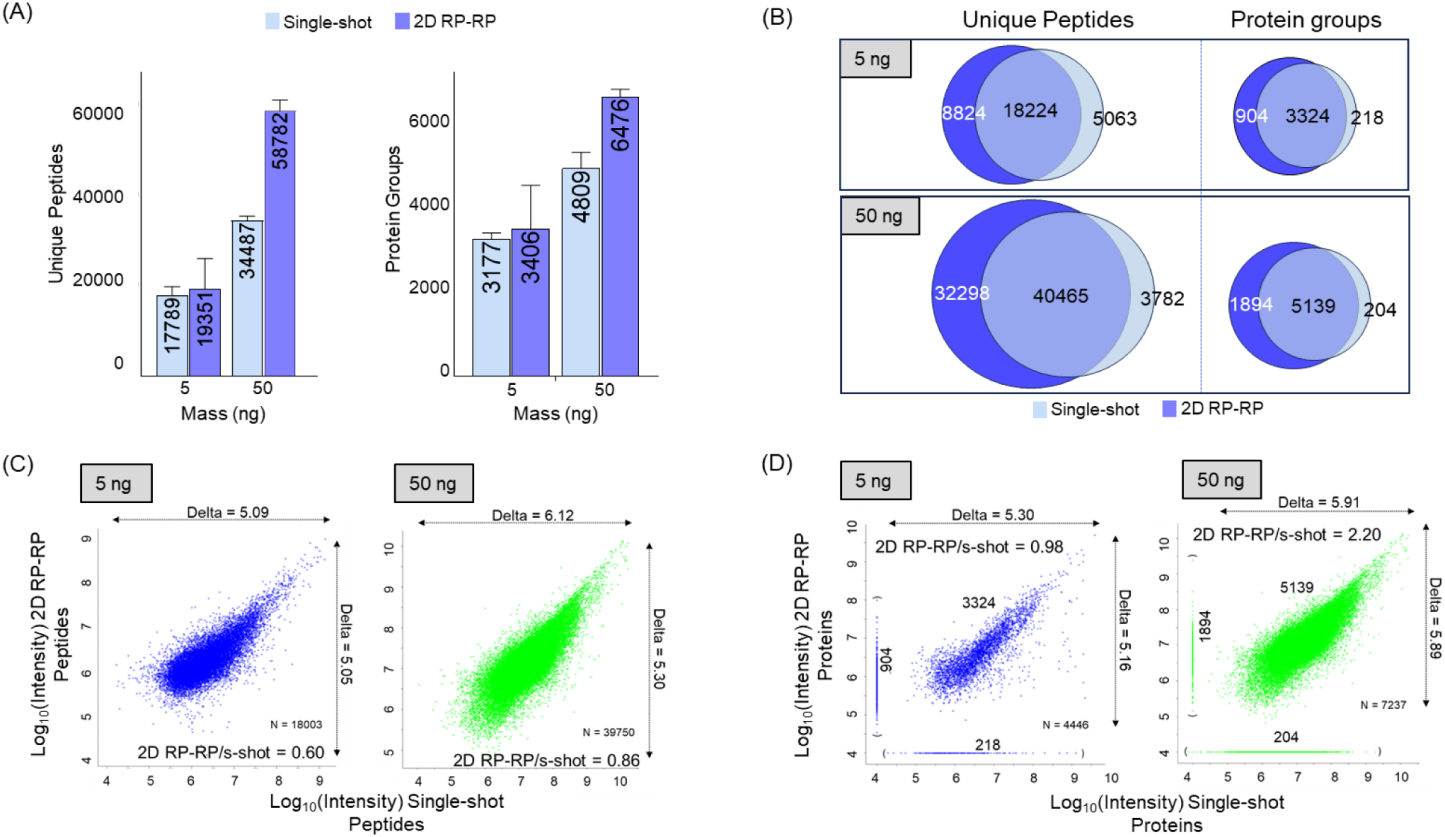
Performance of the 2D RP–RP in the fractionation of 5 and 50 ng of human cell lysate digested peptides. (A) Average number of unique peptides and protein groups identified in single-shot and 2D RP-RP. Error bars correspond to the standard deviation of two replicates. (B) Venn diagrams showing the overlap of the merged numbers of unique peptides and proteins between single-shot and 2D experiments. (C) Scatter plots comparing the mean intensity values of peptides identified in two replicates of 5 and 50 ng single-shot and 2D experiments. Median intensity ratios between single-shot and 2D experiments are shown in the plots. The horizontal and vertical double headed arrows show the dynamic range of the single-shot and 2D experiments at the peptide level. (D) Scatter plots of the proteins quantified in single-shot and 2D experiments. On the X axis (bottom, in brackets), the graphs show the number of proteins only quantified by single-shot, while on the Y axis (left, in brackets), the graphs show the number of proteins only quantified by 2D experiments. The horizontal and vertical double headed arrows show the dynamic range of the single-shot and 2D experiments at the protein level. Raw files were processed using Fragpipe.

The initial analysis suggested that 5 ng (lower limit) and 50 ng of digest for sample loading represent appropriate choices for evaluating sample losses and performance of the 2D system with DDA analysis (performed in duplicated). We saw, on average, a 9% increase in peptides and 7% increase in proteins for the 2D experiment over the single-shot experiment at 5 ng. For the 50 ng digest, we identified, on average, 58 782 peptides and 6476 proteins with the 2D experiment pipeline, corresponding to 70% and 35% increases in both peptides and proteins over single-shot (Figure 3A and Table S2). The vast majority of proteins and peptides observed in the single-shot experiment were also detected in the 2D experiment, with less than 5% being exclusive to the single-shot (Figure 3B). Generation of scatter plots of single-shot versus 2D RP–RP with the peptide and proteins identified with 5 and 50 ng of input material provided insights into performance. The intensity ratio between 2D and single-shot for 5 ng and 50 ng experiments had values of 0.60 and 0.86 at the peptide level, suggesting that, on average, the fractionation experiment had only lost 40% and 14% of the peptide signal, respectively (Figure 3C). The peptides observed exclusively in the single-shot experiment, as expected, correspond to those that have low intensities (Figure S8A). Conversely, the intensity distributions of the peptides observed exclusively by fractionation are low abundant as well (Figure S8B). Clearly, stochastic sampling continues to play a role in data acquisition. As expected, peptide and protein identifications per minute decrease in 2D experiments (Table S3). We observe almost protein signal parity between the two approaches for the 5 ng experiment, but with 50 ng of input material, we observe an increase in reported collective intensity for proteins with 2D compared to single-shot, most likely due to the increased number of peptides identified per protein, which confirms the value of fractionation (Figure 3D). Considering we opted to analyze the same amount by both approaches, it was not surprising that the observed dynamic range does not change significantly when switching to fractionation. It is noteworthy that the 2D fractionation system is capable of extending the experimental dynamic range by increasing sample loading, while with single-shot, increasing loading quickly reaches saturation (Figure 2A). The data suggest that the reason for increased identifications by fractionation is simply complexity reduction, at least for the sample amounts tested. In the single-shot runs, there are many more components competing for ionization, and the charge available per scan (dictated by the AGC) is split across more peptides, reducing signal-to-noise for peak detection.

### Use of DIA and WWA Combined with 2D RP–RP

**Figure 4:**
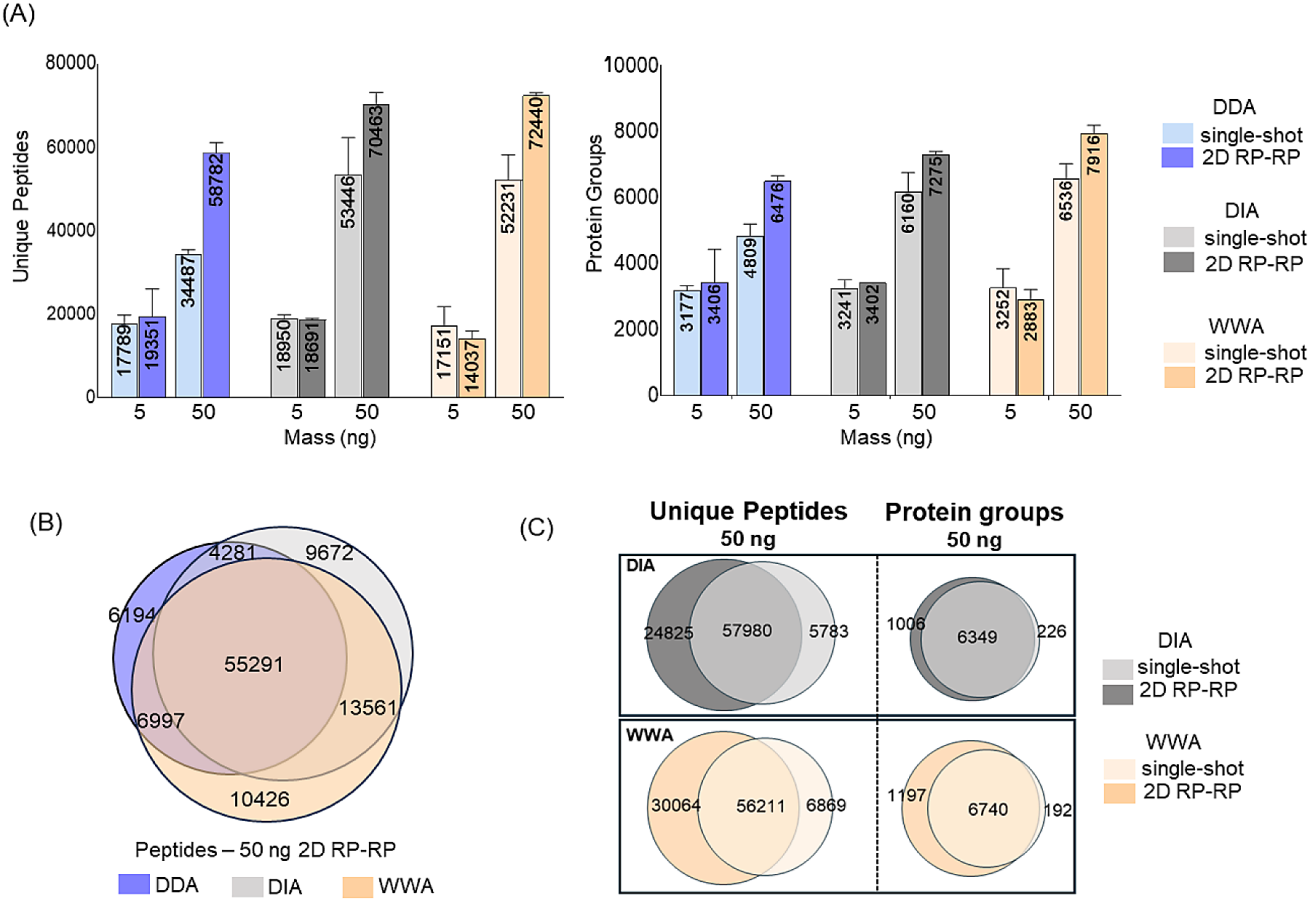
Performance of DIA and WWA in combination with 2D RP–RP. (A) Average number of unique peptides and protein groups identified in single-shot and 2D RP–RP from 5 and 50 ng of human cell lysate digest samples. Error bars correspond to the standard deviation of two replicates. (B) Venn diagram showing the overlap of the merged number of identified peptides across DDA, DIA, and WWA from the fractionation of 50 ng of the starting material. (C) Venn diagrams showing the overlap of the merged number of identified unique peptides and proteins between 50 ng single-shot and 2D experiments using DIA and WWA approaches. Raw files were processed using Fragpipe.

The intensity scatterplots suggest that the advantage of 2D fractionation is the increase in proteome coverage due to the reduction in total signal splitting. Removing the need to select peaks for fragmentation as required by DDA approaches and increasing the AGC for the MS2 might alleviate the precursor selection issue of DDA as shown countless times by the DIA community.^40^ Thus, we repeated the 5 and 50 ng experiments but now using DIA and wide window acquisition approaches (WWA). Table S2 lists the identified peptides using DIA and WWA. The 5 ng experiments were similar in performance to DDA in our hands, with approximately 20 000 peptides and 3000 proteins identified. However, 2D RP–RP combined with DDA appeared to be superior (Figure 4A). Strikingly, both DIA and WWA were substantially better than DDA for the 50 ng single-shot experiment; both approaches nearly matched the performance of the 2D DDA experiment. The 2D RP–RP experiments for DIA showed 32% and 18% increases in peptide and protein identifications, while WWA showed an increase of approximately 39% and 20% at peptide and protein levels compared to the single-shot. Considering it gets progressively more difficult to increase the number of proteins observed as deeper the analysis becomes, such a gain is significant. We also calculated the “completeness” of the identified peptides for both DDA and WWA and observed an increase in missing values for peptide intensity with WWA. Over 50% of identified peptides did not have a corresponding intensity value for the single-shot experiment while that dropped to 33% for 2D. (Figure S9). Comparing the peptides observed across all acquisition approaches, approximately 52% of data are common among all approaches (Figure 4B). Interestingly, uniqueness levels between single-shot and 2D are also observed for DIA and WWA similar to the above DDA experiments (Figure 4C). In our hands, WWA performed similar to DIA^41,42^ suggesting both approaches are compelling choices as alternatives to DDA even when considering 2D RP–RP experiments.

### Investigation of the Use of Enriched Samples and AP-MS with the Fractionation System

**Figure 5:**
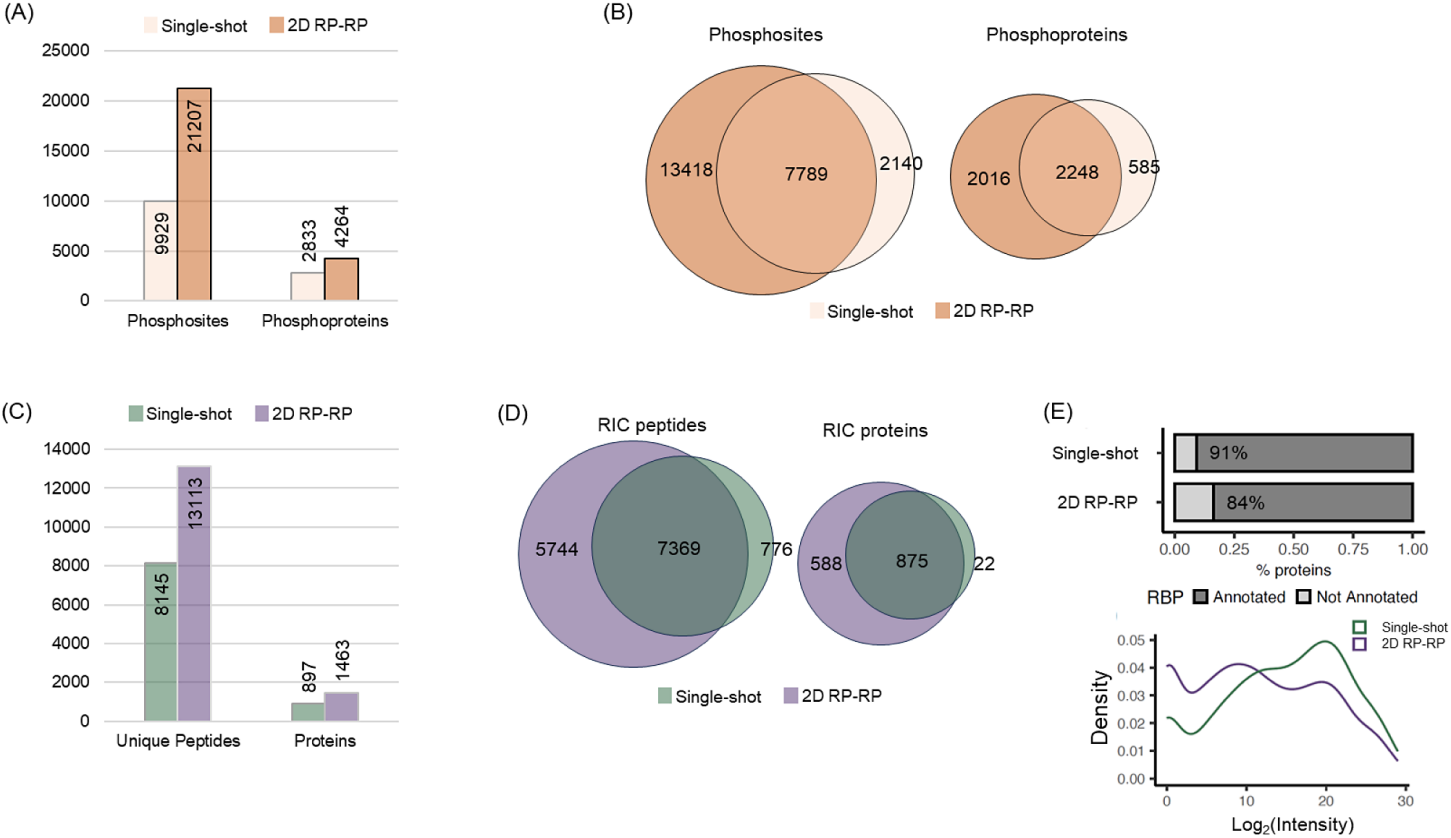
In-depth analysis of subproteomes by 2D RP–RP. (A) Number of phosphosites and phosphoproteins identified in single-shot and 2D experiments in the Zr-IMAC enriched HeLa phosphopeptide sample. (B) Venn diagrams showing the overlap of identified phosphosites and phosphoproteins between single-shot and 2D experiments. (C) Number of unique peptides and proteins identified by single-shot and 2D RP–RP from the analysis of HEK293 RNA-binding proteins isolated by the RNA interactome capture technique. (D) Venn diagrams showing the overlap of identified peptides and RNA-binding proteins between single-shot and 2D experiments. (E) Percentage of annotated RBPs in the set of proteins identified by single-shot and 2D (top) and the intensity distributions of RBPs identified by single-shot and 2D (bottom). Raw files were processed using MaxQuant (for the phosphoproteomics experiment) and Fragpipe (for the RIC experiment).

PTM enrichment is another potential class of experiments that would benefit from a low loss fractionation system. We performed a Zr-IMAC enrichment^24^ on a lysate to isolate the phosphoproteome and subjected 50 μg starting material to a single-shot (60 min gradient) and 2D experiment (30 min gradient, 8 fractions, total 4 h). PTM type experiments disproportionately benefit more from increased sequencing coverage as sites exhibit a peptide-centric property not a protein-centric one, and as expected, we saw a significant increase in the number of phosphosites observed in the 2D RP–RP experiment. Single-shot identified 9929 sites and the 2D experiment identified 21 207 sites (Figure 5A and Table S4). Overlap between single-shot and 2D suggested a loss of a significant number of sites (2140 phosphosites) (Figure 5B). The exclusive sites observed for single-shot and 2D are in the lower intensity range; nevertheless, the 2D approach substantially identifies more new unique sites than those that are lost (Figure S10).

We also tested for a protein enrichment experiment by applying the 2D system to an RNA interactome capture (RIC)^25,43^ experiment, which enriches for RNA-binding proteins in cultured cells (HEK293 cells in this case). RIC employs irradiation of cell monolayers with ultraviolet (UV) light to promote “zero distance” RNA-to-protein cross-links, followed by the capture of protein–RNA complexes using oligo(dT) magnetic beads and elution by heat and RNases. Single-shot proteomic analysis revealed 897 RBPs. Strikingly, RBP identification increased to 1463 when our 2D approach was applied, with over 98% of RBPs identified in the single-shot also being identified in the fractionated samples. Over five hundred additional RBPs were identified in the 2D fractionated run over the single-shot experiment, suggesting a substantial increase in depth (Figure 5C,D and Table S4). More than 84% of proteins identified in the fractionated run have been previously identified as RBPs (defined as occurring in 3 or more data sets in RBPbase^25^), which is slightly lower than with the single-shot analysis (91%, Figure 5E, top). The lower hit rate, therefore, suggests a cautious approach to be taken for assigning the RBP status and validated by orthogonal methods. Alternatively, it indicates that 2D may enable the identification of low abundance or substoichiometric RBPs that conventional single-shot approaches miss. Supporting this, 2D fractionation increased the coverage of proteins classified as RBPs that are expressed at low levels in cells (Figure 5E, bottom). This might reflect the identification of low-abundance RBPs that are often missed in the absence of sufficient depth. Altogether, these results highlight the power of our 2D fractionation method to improve our understanding of the cellular proteome.

## Conclusions

We have developed a versatile and cost-effective fraction collector that can be coupled to a nano-LC to enable deep proteome coverage from low amounts of material. In combination with DDA LC-MS analysis, the use of our fractionation system led to a substantial increase in peak capacity and identification rates over single-shot experiments for the analysis of human cell lysate, demonstrating superior proteome coverage using only 5 ng of digest, with performance increasing with increasing amounts of material. We envision that our 2D setup can be readily applied to various DDA centric proteomics workflows and could be particularly useful for isobaric multiplexed experiments (e.g., TMT) applied to small cell populations, where sample availability is the issue. The application of DIA and WWA to fractionated samples also suggested value in the use of fractionation with the 2D WWA experiment, allowing identification of approximately 8000 proteins from just 50 ng of material. Furthermore, the low loss 2D system demonstrably benefits phosphoproteomics and pull-down experiments, expanding the coverage of both subproteomes relative to single-shot approaches.

## Supporting Information Available

Sample preparation, phosphopeptide enrichment, RNA interactome capture, off-line high-pH reversed-phase nanofractionation system, nano-liquid chromatography and mass spectrometry, and data analysis; nanofractionation system overview, showing the Evosep One connected to the 3D-printer-based fraction collector and the 3D-printed column bracket and platform (Figure S1); Evosep One HP pump pressure profile using a commercial EV-1106 and an in-house packed column at high and low-pH LC-MS (30SPD) (Figure S2); column stability evaluation under basic conditions showing the retention time alignment of five peptides identified in Expi293F digest by high-pH LC-MS/MS (Figure S3); orthogonality plot showing the retention times of Expi293F peptides analyzed by high and low-pH LC-MS/MS using the 30SPD method (Figure S4); peptide recovery performance of the 2D RP–RP system showing the comparison of peptide intensities between nonfractionated and fractionated/pooled human cell lysate digested peptides resuspended in 5% FA (A) or 5% FA/0.015% DDM prior to fractionation (Figure S5); histograms showing the observed frequency of peptides detected in one or multiple fractions for 50 ng fractionation experiments using DIA and DDA (with “match between runs” feature enabled) modes (Figure S6); base-peak chromatograms of the single-shot (top) and 2D RP–RP (bottom) analysis of 50 ng of human cell lysate digested peptides, showing the median values of fwhm and the theoretical peak capacities (Pc) of single-shot and 2D experiments (Figure S7); histograms showing the intensity distributions of the entire set and exclusively identified peptides in single-shot and 2D RP–RP LC-MS/MS DDA analyses of 5 and 50 ng of Expi293F digest (Figure S8); number of peptides identified by single-shot and 2D RP–RP using DDA and WWA approaches showing the numbers of valid and missing values (Figure S9); histograms showing the intensity distributions of the entire set and exclusive phosphosites identified by single-shot and 2D RP–RP LC-MS/MS DDA analyses from the Zr-IMAC enriched HeLa phosphopeptide sample (Figure S10) (PDF)
Identified peptides categorized by sample amount in single-shot and 2D RP–RP DDA experiments (XLSX)
Peptides and proteins identified using DDA, DIA and WWA in 5 and 50 ng single-shot and 2D RP–RP experiments (XLSX)
Identification performance of 5 and 50 ng DDA, DIA and WWA single-shot and 2D RP–RP experiments (XLSX)
Phosphosites and RIC peptides identified in single-shot and 2D RP–RP experiments (XLSX)
